# Le pemphigus végétant: une rare dermatose des plis

**DOI:** 10.11604/pamj.2013.15.134.2411

**Published:** 2013-08-14

**Authors:** Mariame Meziane, Fatima-zahra Mernissi

**Affiliations:** 1 Service de Dermatologie, CHU Hassan II Fès, Maroc

**Keywords:** Pemphigus végétant, dermatose des plis, dermatose bulleuse auto-immune, pemphigus vegetans, dermatosis of the folds, autoimmune bullous dermatosis

## Image en médecine

Une patiente de 42 ans, sans antécédents pathologiques notables consultait pour l'apparition depuis 6 mois de lésions cutanées ombilicale et sus pubienne traitées localement et à plusieurs reprises sans amélioration. L'examen clinique retrouvait une patiente en bon état général avec des lésions végétantes, suintantes, malodorantes et violacées au niveau de l'ombilic et au niveau sus pubien. Le signe de Nicolsky était négatif et il n'existait pas d'autres anomalies cutanées ou muqueuses. La biopsie cutanée mettait en évidence une acanthose avec des bulles intraépidermiques suprabasales, l'immunofluorescence directe montrait un dépôt intradermique en résille d'IgG et de C3. Le diagnostic de pemphigus végétant a été retenu. Un traitement par la corticothérapie orale à raison de 1,5mg/kg/j et par l'Azathioprine à 2mg/kg/j a été débuté avec cicatrisation complète des lésions après 3 semaines. Le pemphigus est une dermatose bulleuse auto-immune rare, la forme végétante en est une forme exceptionnelle. Elle se manifeste par des lésions bourgeonnantes en relief, rarement par des bulles et se localise essentiellement au niveau des plis avec risque de macération et de surinfection.

**Figure 1:**
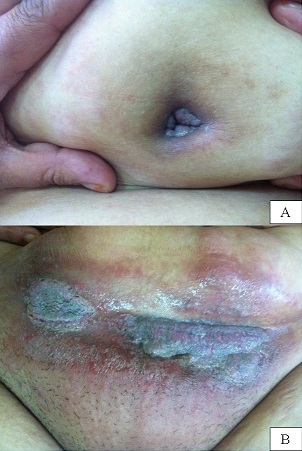
A) Lésions mamelonnées violacées de siège ombilical; B): Aspect de placard végétant violacé macéré au niveau sus pubien

